# Innovation and entrepreneurship programs in US medical education: a landscape review and thematic analysis

**DOI:** 10.1080/10872981.2017.1360722

**Published:** 2017-08-09

**Authors:** Blake A Niccum, Arnab Sarker, Stephen J Wolf, Matthew J Trowbridge

**Affiliations:** ^a^ Department of Emergency Medicine, University of Virginia, Charlottesville, VA, USA

**Keywords:** Entrepreneurship, innovation, design thinking, medical education, changing healthcare system

## Abstract

**Background:** Training in innovation and entrepreneurship (I&E) in medical education has become increasingly prevalent among medical schools to train students in complex problem solving and solution design.

**Objective**: We aim to characterize I&E education in US allopathic medical schools to provide insight into the features and objectives of this growing field.

**Design**: I&E programs were identified in 2016 via structured searches of 158 US allopathic medical school websites. Program characteristics were identified from public program resources and structured phone interviews with program directors. Curricular themes were identified via thematic analysis of program resources, and themes referenced by >50% of programs were analyzed.

**Results**: Thirteen programs were identified. Programs had a median age of four years, and contained a median of 13 students. Programs were led by faculty from diverse professional backgrounds, and all awarded formal recognition to graduates. Nine programs spanned all four years of medical school and ten programs required a capstone project. Thematic analysis revealed seven educational themes (innovation, entrepreneurship, technology, leadership, healthcare systems, business of medicine, and enhanced adaptability) and two teaching method themes (active learning, interdisciplinary teaching) referenced by >50% of programs.

**Conclusions**: The landscape of medical school I&E programs is rapidly expanding to address newfound skills needed by physicians due to ongoing changes in healthcare, but programs remain relatively few and small compared to class size. This landscape analysis is the first review of I&E in medical education and may contribute to development of a formal educational framework or competency model for current or future programs.

**Abbreviations**: AAMC: American Association of Medical Colleges; AMA: American Medical Association; I&E: Innovation and entrepreneurship

## Introduction

In a rapidly changing healthcare system, students entering US medical schools today have educational needs and goals unmet by the current medical education system [1]. The ongoing emergence of disruptive technologies and systemic changes in personalized medicine, regulations, and reimbursement models present imminent and uncertain challenges for physicians beyond the basic and clinical sciences comprising traditional medical education [1–3]. Solving these new challenges will require not only clinical mastery, but also the ability to design, develop, and implement patient-centered solutions to complex problems [2,4]. Both the American Association of Medical Colleges (AAMC) and American Medical Association (AMA) recognize systems-level problem solving as a basic requirement of medical graduates, but there is little consensus regarding the key skills and knowledge needed to achieve this goal [5,6]. Innovation and design-related education has emerged as a potential source of novel teaching methods and concepts that address this need for problem-solving skills in medical education.

**Figure 1.**
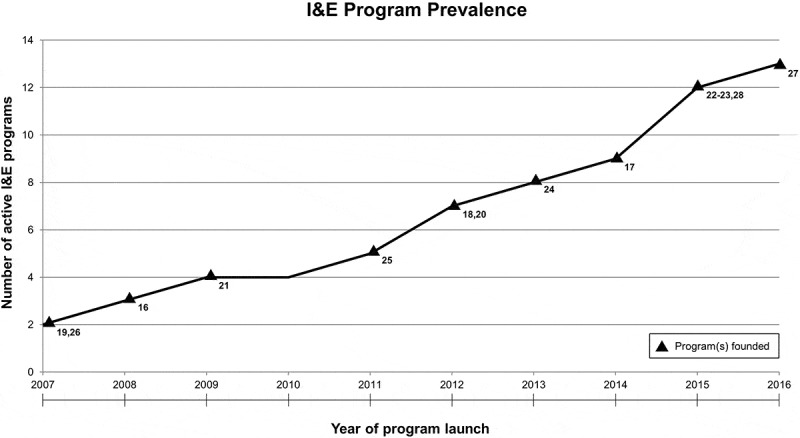
I&E program prevalence at US allopathic medical schools from 2007-2016. As demonstrated in the figure, medical school I&E programs increased nearly every year during this period. The symbol ▲ denotes years in which the identified I&E programs were founded. The corresponding program names and associated institutions are listed in Table 1.

**Figure 2.**
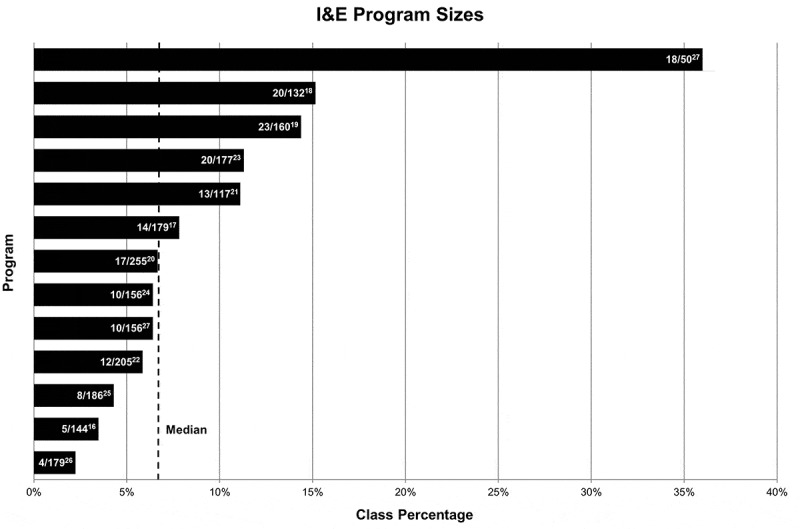
I&E program sizes (i.e. number of participating students) as a percentage of total medical school class size. Program sizes vary greatly across the identified programs. Absolute values used to calculate these percentages are provided as average yearly program size divided by average medical school class size. Numerical superscripts correspond to the I&E programs and associated institutions listed in Table 1.

A number of medical schools have approached the issue of training students to engage in complex problem solving using formal curricular programs. Taking many shapes and sizes, these innovation and design related programs generally fall under the umbrella terms of *innovation and entrepreneurship* (I&E). Historically, I&E has been taught in undergraduate courses focused on business or product development, but is now a common field of study in graduate business, engineering, and design programs [7–10]. As I&E programs begin to filter into medical education, it is compelling to understand how they supplement medical curricula, and what medical education as a whole may draw from them in the future. Certainly, physicians will be called upon to help solve the current and future challenges in the healthcare system. As physicians take on leadership roles and develop products and solutions to complex problems, I&E education may help inform these endeavours.

I&E is a new term to medical education, and determining a specific and concise definition for medical I&E education is challenging. Certain definitions, such as the one from The Entrepreneurship Competence Framework, generally define the ‘entrepreneurship’ competency as the ability to turn ideas into actions [11]. Other competency models addressing both innovation and entrepreneurship include structured problem solving, needs analysis, social orientation, and technical skills such as financial analysis [12–14]. For the purpose of this study, we define ‘innovation and entrepreneurship’ education in the most general sense, as the process of learning how to generate ideas and translate these ideas into a defined target, such as a product or initiative. In doing so, we aim to identify the key skills and concepts that will help medical students engage in complex problem solving in their future careers.

Despite recent integration of I&E education into the curricula of several medical schools, no comprehensive overview of these programs has been published. Resources from organizations such as the AAMC and AMA have yet to address I&E or specific program curricula. The objective of this study is to provide a landscape analysis of I&E programs at US allopathic medical schools. We also aim to provide educators with the foundational resources to inform I&E curricula at their own institutions. The analysis will address the following four research questions:

1. What I&E programs exist at US allopathic medical schools?
2. What are the characteristics of medical I&E programs?
3. What are common topics addressed by medical I&E programs?
4. What are common teaching methods used by medical I&E programs?

## Methods

We conducted a mixed-methods study in 2016 consisting of three phases: identification of I&E programs, evaluation of program characteristics, and thematic analysis for common curricular content.

### Ethical considerations

This study was conducted using public data (e.g. webpages, downloadable informational materials) and interview responses from program directors regarding their I&E programs. Program directors reported on aspects of their curricula as key informants and content experts, and did not provide any personal data. Analysis was performed only on public data. Data from interviews was used to report on program characteristics and teaching methods – no additional analysis or generalization was performed. As such, this study did not meet the requirements for human subject review per Institutional Review Board protocol, and ethical approval was not required.

### Identification of I&E programs

We performed a systematic search of all 158 US allopathic medical school curriculum websites in order to identify relevant I&E programs. Key search words employed to locate programs included: *innovation, entrepreneurship, design, technology, invention, concentration, track, curriculum*, and *capstone project*. For inclusion in our study, programs needed to have documented curricular goals consistent with above mentioned I&E principles, be officially sanctioned by their medical school, and not exist as an isolated fourth year elective. To identify additional programs missed by our search, directors of included programs reviewed the compiled list and were offered the opportunity to add unidentified programs.

Of note, we initially sought to identify programs via the AAMC Curriculum Inventory and Reports (CIR), a public repository for medical school curriculum data. However, we discovered that the AAMC CIR did not mention ‘innovation’ or ‘entrepreneurship’. Moreover, the data available via the CIR was aggregate in nature and did not enable us to discern sufficiently detailed information about individual programs. Thus, we chose instead to identify I&E programs by the aforementioned website search and program director referral.

### Program characteristics

After initial review of all public data, we created a survey-based data collection instrument pertaining to program characteristics. We performed data abstraction from each of the included program’s publically available resources, and then contacted program directors in order to complete individual data collection instruments where needed. Specific program characteristics collected include: age (i.e. number of years that the program has been in place), size (i.e. number of students in proportion to medical school class size), core faculty members (i.e. number of core faculty, degrees earned by faculty), types of formal recognition awarded to students by programs, and structure (i.e. program duration, summer experience requirement between the first (M1) and second (M2) years of medical school, capstone project requirement). For the purposes of our survey, core faculty members represent those that identify themselves as ‘core faculty members’ for the program.

### Thematic analysis

Common curricular themes were identified via inductive thematic analysis of publicly available program resources. We employed grounded theory in ethnography to guide our analysis, allowing us to: compare data with data from the beginning, not after all data were collected; to compare data with emerging themes; and, to demonstrate relations between concepts and themes [15]. Two data abstractors (B.N. and A.S.) used an open-ended data collection instrument accommodating program text to collect qualitative information from each identified program. Using an inductive approach, data was analyzed for main ideas and reduced to underlying themes through iterative discussions by the research team. Themes referenced by the majority of programs (>50%) were conserved for analysis. Discrepancies in conserved themes resulting from use of slightly differing terminology between programs were consolidated based on consensus among the research team. Conserved themes were categorized into educational (i.e. related to specific topics) and teaching method themes.

## Results

### Identification of I&E programs

Thirteen of the existing 158 (13/158; 8.2%) US allopathic medical schools had identifiable programs that met our inclusion criteria (see Table 1). Program directors of all identified programs (13/13; 100%) were successfully contacted. No additional programs were identified by program director referral.

**Table 1.** I&E programs at US allopathic medical schools, as identified via 2016 structured internet search.

Medical school	I&E program name
Brown University Warren Alpert Medical School (BROWN)	Concentration in medical technology, innovation and entrepreneurship[16]
George Washington School of Medicine and Health Sciences (GW)	Clinical practice innovation and entrepreneurship track[17]
New York University School of Medicine (NYU)	Health systems innovation and policy concentration[18]
Northwestern University Feinberg School of Medicine (NU)	NUvention: Medical[19]
Thomas Jefferson University Sidney Kimmel Medical College (TJU)	College within the college design track[20]
University of Arizona College of Medicine (UA)	Leadership and innovation in healthcare distinction track[21]
University of Illinois College of Medicine at Chicago (UIC)	Innovation medicine program[22]
University of Michigan Medical School (UM)	Innovation and entrepreneurship path of excellence[23]
University of Pennsylvania Perelman School of Medicine (UPENN)	Certificate in healthcare management, entrepreneurship, and technology[24]
University of Southern California Keck School of Medicine (USC)	Health, technology and engineering program[25]
University of South Florida Morsani College of Medicine (USF)	Innovation, entrepreneurship and business in medicine scholarly concentration[26]
University of Texas at Austin Dell Medical School (UT)	Health care innovation & design distinction[27]
University of Virginia School of Medicine (UVA)	Human-centered design and medical innovation program[28]

### Program characteristics

The following program characteristics provide an overview of basic features of identified I&E programs:

1. *Age*: The median age of programs from inception was four years. As shown in Figure 1, the oldest programs were nine years old. Three programs welcomed their first cohort of students in 2015, and one program welcomed its first cohort in 2016.
2. *Size*: Programs accepted a median of 13 new medical students per year. As shown in Figure 2, the smallest program accepted approximately four students per year, while the largest program accepted 20–25 students per year. With respect to medical school class size, the median program comprised 7% of the student body, with a range of 2–36% of total class size.
3. *Core faculty*: Programs were typically led by a small group of core faculty members from a wide array of backgrounds. The average program had three core faculty members. The program with the largest number of core faculty had nine faculty members. However, this program also had significant involvement from student leadership. Degrees associated with core faculty members across programs included: MD, PhD, MBA, MPH, BS in BME, RN, and MSCE.
4. *Formal recognition awarded*: All surveyed programs awarded at least one form of formal recognition to program graduates. Students received recognition in their medical student performance evaluation (MSPE) or ‘dean’s letter’, medical school transcript, medical school diploma, and/or via a program-specific certificate.
5. *Program structure*: Most programs (9/13; 69%) spanned all four years of medical school. The remaining programs included a year-long program for M1 students, a year-long program for fourth year (M4) students, a six month program for M4 students, and a three year program starting in the second year.
  In four year programs, the transitions in program structure generally mirrored transitions in core curriculum. The pre-clerkship periods traditionally taking place during M1 and M2 were comprised of monthly didactics and workshops, with some programs using weekends during this time to hold more intensive workshops focused around single subject areas.
  Of the programs spanning more than one year, seven (7/10; 70%) required completion of a program-related experience during the summer between M1 and M2. These summer experiences included research projects, internships, quality improvement initiatives, device development, and entrepreneurial ventures. The survey results did not show common criteria for which types of experiences can meet summer requirements.
  Programs tended to reduce required activities during the core clinical clerkships, with 3/13 (23%) reporting informal group discussions or journal clubs during this time. All programs spanning greater than one year (10/13; 77%) required completion of a capstone project during M4 where students work with faculty members to pursue a research question or develop a solution to a clinical or quality improvement need. All programs required a presentation or report at the culmination of the project, with some programs including even more concrete endpoints. For example, one program required students to file a provisional patent by the end of their experience. Time dedicated to the capstone project ranged from several months to an entire year during M4.

### Thematic analysis

Public resources were retrieved for 13/13 (100%) programs. Extractors identified a total of 40 concepts, which were consolidated into 20 themes. Seven educational (7/20; 35%) and two teaching method (2/20; 10%) themes were conserved.

#### Educational themes

1. *Innovation* (12/13; 92%): Innovation was consistently identified as a tool for physicians desiring to create impact through the translation of their ideas into products/devices, businesses, health policies, or system-wide improvements. Example skills listed as related to this educational theme include idea creation [16], navigating complex environments [28], and interdisciplinary problem solving [22].
2. *Entrepreneurship* (11/13; 85%): Programs defined entrepreneurship as the commercialization of products/solutions into viable business ventures. Entrepreneurship pertained to both small scale ventures (i.e. start-up companies) [17] and ventures within larger organizations (i.e. large corporations, health systems, or government) [19]. Example skills listed as related to this educational theme include business planning [17], executive leadership [16], interdisciplinary teamwork [22], and financial management [19].
3. *Leadership* (10/13; 77%): Programs cited leadership as a tool for achieving organizational success. However, there was no consensus among programs about the specific definition of successful leadership or its unique role within I&E. Example skills listed as related to this educational theme include ‘[understanding] leadership styles, change management, conflict resolution, inter-professional team dynamics, [and] use of organizational management tools’ [21].
4. *Technology* (10/13; 77%): The understanding of technology was defined across programs as the ability to adeptly use and develop medical products, devices, and/or digital technologies. Programs consistently suggested that understanding of technology is necessary for the translation of potential solutions into clinical application. Example skills listed as related to this educational theme include interdisciplinary collaboration [25], product development [22], needs identification [19], and prototype design [19].
5. *Healthcare systems* (8/13; 62%): The understanding of healthcare systems was defined as the ability to understand the various interactions between stakeholders in healthcare. There were varying definitions of a healthcare system, ranging from individual community health systems to the entire US healthcare system [17,21]. However, it was stated across programs that a functional knowledge of healthcare systems is required to develop innovative products/solutions. Example skills listed as related to this educational theme include advocacy [18] and systems-based practice [20].
6. *Business of medicine* (7/13; 54%): Working knowledge of finance and reimbursement was cited by several programs as a valuable skillset. There was a wide range of applications of this educational theme, including a general understanding of financial reimbursement [21], the application of new reimbursement models such as value-based care [19], and the creation of financial models to justify investment opportunities [19].
7. *Enhanced adaptability* (7/13; 54%): The need for enhanced adaptability was mentioned in the context of preparing future physicians for a changing healthcare system. Specifically, programs sought to prepare students for changes in the technological, financial, and regulatory environment [17,28]. The application of enhanced adaptability was cited as ranging across commercial, academic, and/or clinical pursuits. There were no specific skills listed as related to this educational theme.

#### Teaching method themes

1. *Active learning* (13/13; 100%): All programs included elements of active learning, helping students practice the skills-based components of innovation. Active learning manifested in the form of group workshops, prototyping sessions, hackathons, field trips to innovative companies, M4 capstone projects, internships, and immersion experiences.
2. *Interdisciplinary teaching* (11/13; 85%): Nearly all programs taught their students in interdisciplinary settings. Programs incorporated students and faculty from medical, engineering, science, business, and/or law backgrounds, and identified interdisciplinary collaboration as essential to healthcare innovation, particularly in the translation of medical technologies into effective clinical solutions.

## Discussion

Our mixed methods study of US allopathic medical schools identifies a small but rapidly growing presence of I&E programs in medical education. These programs are advancing student readiness for today’s complex healthcare environment by covering novel educational themes with active, interdisciplinary pedagogy.

These programs were comprised of student subsets representing less than 15% of a given medical school class, were led by several core faculty members from diverse career backgrounds, and awarded graduating students with at least one form of formal recognition. Programs varied with respect to structure, though the majority spanned greater than one year and required completion of a capstone project.

Thematic analysis revealed that programs addressed many of the same competencies and employed similar teaching methods. The majority of programs taught concepts pertaining to innovation and its application in entrepreneurship, leadership, technology, healthcare systems, and the business of medicine. Programs cited that cultivation of these competencies will provide enhanced adaptability within a changing healthcare landscape. Programs helped students hone these competencies through active learning techniques, including workshops, prototyping sessions, and capstone projects. Experiences also tended to be interdisciplinary, in that students and faculty were typically from diverse educational and professional backgrounds. Though these categories provide a sense of general curricular goals, none of the programs publicly proposed a formal competency model. In addition, the results show a lack of consensus regarding the specific skill sets required to reach competency within the educational themes. This lack of connection to skills is best illustrated in the themes of healthcare systems and enhanced adaptability.

Interestingly, our study revealed two exciting examples of program graduates applying their I&E education beyond the classroom. In 2012, four Northwestern graduate students launched a medical device company, Briteseed, which markets a surgical device the students invented while participating in *NUvention: Medical* [27]. Likewise, in 2013, four graduates from the University of Southern California’s *Health, Technology and Engineering Program* launched StemSurgical Inc., a medical device company that designs high throughput bone marrow harvesting tools [28].

We also identified a number of novel education initiatives which have otherwise not been noted in other areas of medical education. As an example, the field of design thinking, a problem solving methodology that utilizes user-centric strategies such as ideation and rapid prototyping to solve complex problems, has found its way into a growing number of I&E programs [18,23,26,29]. These new concepts and skills are widely applicable beyond the commercialization of products, and may over time find a greater role in I&E education, and perhaps in medical education as a whole.

The study had several limitations. To the best of our knowledge, no resource has previously compiled a list of medical school I&E programs, and thus we relied upon our described search techniques and insight from other program directors to avoid omitting eligible programs. Therefore, it is possible that additional programs exist at allopathic medical schools that were missed by our search methods. In addition, we limited our analysis to allopathic medical schools, so our findings cannot be extrapolated to I&E programs that may exist at osteopathic medical schools. Our definition of I&E programs also excluded individual courses or more informal offerings, which likely represent another important facet of I&E education. Finally, our thematic analysis relied on publicly accessible information, and it is possible that many programs have changed curricula or policies since publishing this information.

We hope to increase transparency and collaboration among the cited programs, and to provide educators at medical schools lacking I&E programs the foundational resources to launch similar programs at their own institutions. Additionally, although all programs cited in our study have descriptions available online, prospective medical students currently lack an efficient means by which to learn which medical schools offer I&E programs. We aim to empower prospective medical students interested in I&E with insight into this emerging educational niche.

## Conclusion

The landscape of I&E programs among US allopathic medical schools is rapidly expanding to address the newfound needs placed on physicians by recent and ongoing changes within healthcare. Considering that these programs aim to address aspects of medicine that all physicians will encounter, we propose further study of I&E education and how it may eventually integrate into the core medical school curriculum. Efforts to increase transparency and collaboration among existing I&E programs should promote the optimization and expansion required to eventually enable the widespread integration of I&E into medical education.
